# Supplementary figures and images for: Rapid progression is associated with lymphoid follicle dysfunction in SIV-infected infant rhesus macaques
Source: PLoS Pathog. 2021 May 7;17(5):e1009575. doi: 10.1371/journal.ppat.1009575 (PMC8133453; doi:10.1371/journal.ppat.1009575)

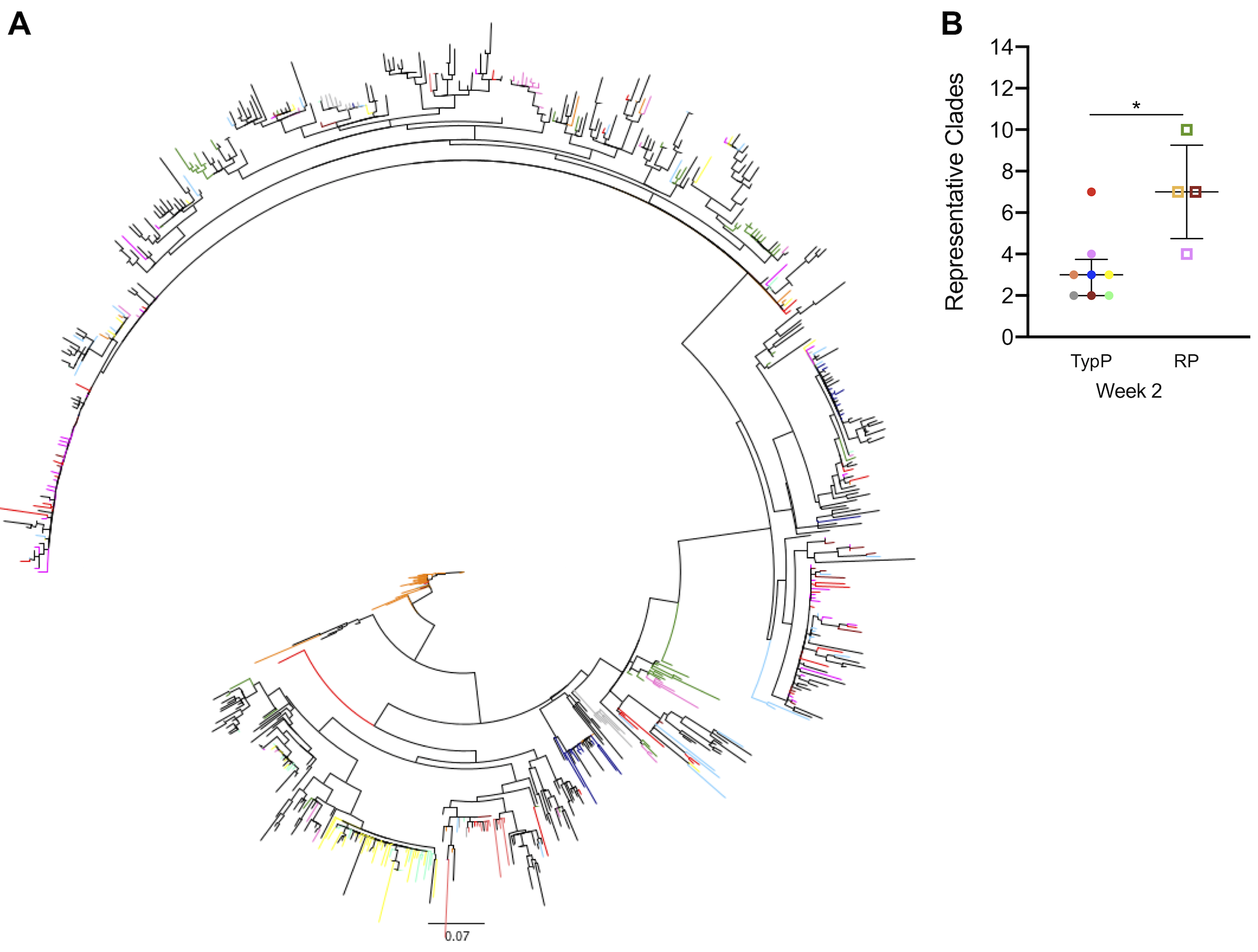

Supplement: S1 Fig — Phylogeny of macaque-derived plasma V1V2 variants isolated at 2 weeks post-infection(colored) and variants within challenge stock (black) (A). The number of clades in which each macaque-derived variant was represented was enumerated and the numbers of representative clades were compared between variants from TypP and RP macaques (B). Phylogenies were constructed using maximum likelihood with a GTR substitution model using the challenge stock consensus sequence. Statistical tests used to compare infant groups were carried out as described in the methods * = p<0.05. Error bars are shown as either mean with standard deviation or median with interquartile range based on data distribution. (TIF) [file ppat.1009575.s001.tif]

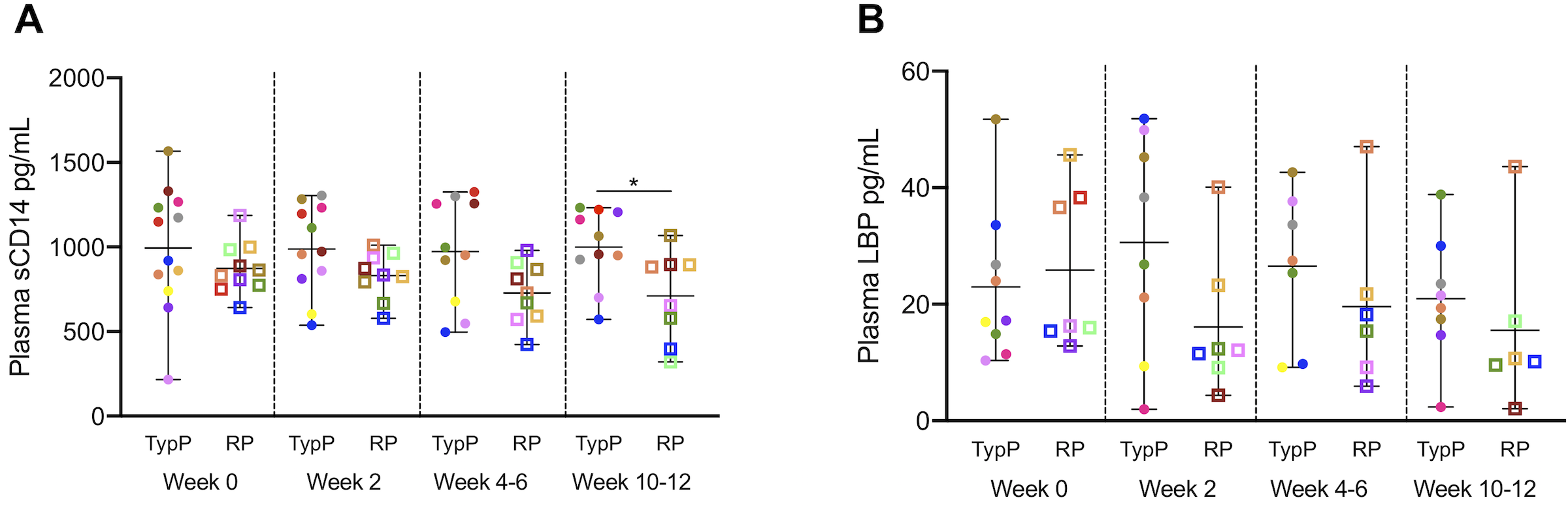

Supplement: S2 Fig — Plasma sCD14(A) and LBP(B) concentrations were measured by ELISA at timepoints prior to and following SIV infection in TypP and RP infants. Statistical tests used to compare infant groups were carried out as described in the methods * = p<0.05. (TIF) [file ppat.1009575.s002.tif]

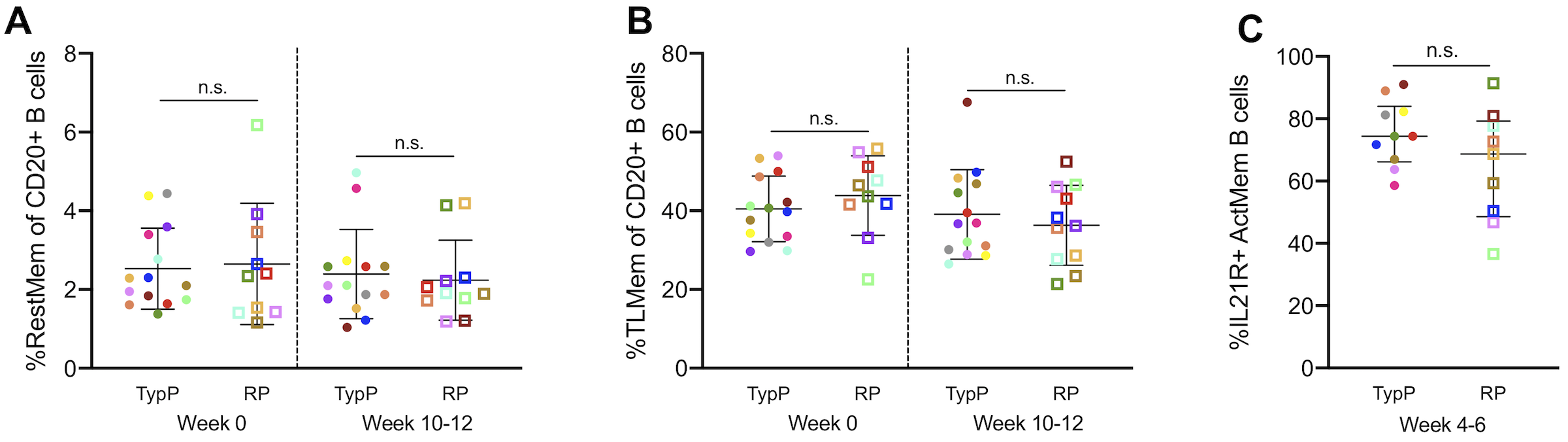

Supplement: S3 Fig — Proportions of memory B cell subsets in TypP infants (closed circles) and RP infants (open squares). Percentages of resting memory B cells (RestMem, CD21+, CD27+) and Tissue-Like memory B cells (TLMem, CD21-,CD27-) were evaluated within the total B cell (CD20+) population in PMBC (A+B). The percentage of Activated Memory B cells (ActMem, CD21-, CD27+) was measured expressing the IL21 receptor (IL-21R) (C). Statistical tests used to compare infant groups were carried out as described in the methods. Error bars are shown as either mean with standard deviation or median with interquartile range based on data distribution. (TIF) [file ppat.1009575.s003.tif]
