# Supplementary material for: Rapid progression is associated with lymphoid follicle dysfunction in SIV-infected infant rhesus macaques
Source: PLoS Pathog. 2021 May 7;17(5):e1009575. doi: 10.1371/journal.ppat.1009575 (PMC8133453; doi:10.1371/journal.ppat.1009575)
Supplement: Attachment — Submitted filename: 2021_ReviewerResponses_03082021.docx [file ppat.1009575.s004.docx]

We would like to thank the reviewers for the helpful comments and critique of our manuscript.  Responses to individual comments are below.

**Reviewer #1:**

*1. In the abstract the authors indicated “had similar CD4 T Cell depletion”, however this is not supported by figure 3 panel C, please rephrase the sentence.*

Thank you for pointing out this oversight. This line has been corrected to reflect our data.

*2a. In the introduction, lines 74-76 the authors mentioned the relationship between viremia and type I IFN, a reference should be also included (Campillo-Gimenez L, J Virol. 2010 Feb;84(4):1838-46. doi: 10.1128/JVI.01496-09). Indeed, a clear relationship between pDC, IFN levels and monkey pathogenesis is described in this manuscript.*

2b. *Lane, 77-78, please also include these two references that demonstrated the link between the extent of cell death, B cell follicle activation and abortive Ig responses (Monceaux V, AIDS. 2003 Jul 25;17(11):1585-96. doi: 10.1097/00002030-200307250-00002.; Cumont MC, J Virol. 2008 Feb;82(3):1175-84. doi: 10.1128/JVI.00450-07). These could be also introduced in the discussion and are in line with the observations made in this manuscript.*

*2c. Line 221, the authors assessed the link between pDC and IFN in infant monkeys that perfectly fit with the notion described by Campillo-Gimenez L (J Virol 2010).*

*2d. Lines 286-291, In fact Tfh cells are localized at the border of B cell follicle (see Moukambi Plos pathogens and Moukambi F, Mucosal Immunol. 2019 Jul;12(4):1038-1054. doi: 10.1038/s41385-019-0174-0), and perfectly fit with the observations of viral replicative cells and MX1 expression in infant monkeys. Furthermore, in this manuscript (Mucosal immunology), the authors also addressed the expression of CXCL13 in relationship with follicle architecture and therefore should be discussed. Indeed, instead to do a list of results (line 302-307) this part in infant merits to be further discussed.*

The reviewer’s comments pertaining to a number of published studies that were not adequately referenced in first submission prompted us to look carefully at our introduction and discussion sections of the manuscript. We have concluded that these references are all very important in understanding our work in context of findings that have come before. Therefore, we have made sure to include the references as suggested, while also taking this opportunity to alter the text in both the introduction and in the discussion to allow for a more robust discussion of the papers being referenced. We would like to thank the reviewer for these suggestions. The revised sections can now be found at lines 67-90, 292-299 and 315-317.

*3. Lane 79-80 please add “infant” in the sentence.*

We have added the word “infant” to this line.

*4. The authors proposed that the absence of depletion is related to an expansion of naive T cells (line 144). If the authors have the opportunity to perform additional staining, this will greatly improve the sentence. Thus, the term “unexpectedly” is not appropriate in this context (line 161).*

We agree and have altered the phrasing of line 144 has been edited to summarize previous work identifying an expansion of naive CD4 T cells, without speculating as to the phenotype of CD4 T cells in our study. Additionally, we have removed the term “unexpectedly” from line 161. Unfortunately, due to limited sample availability (because these are infants) we are unable to do more staining. This sentence can now be found at lines 151-152

*5. In the discussion, the authors stated that “aberrant IFN-driven immune activation contributes to B cell dysfunction”. However, there is no immune activation in RP whereas IFN levels were higher. The sentence should be deleted whereas the observations are consistent with previous reports. Thus, in lane 266, the authors should discuss these manuscripts (Campillo-Gimenez L and Cumont) since they are in line with their observations.*

We agree, this is an important point that required clarification. The term “immune activation” has been deleted, as this term typically refers to T cell activation, which was not increased in the RP infants. To be more accurate, our findings support a model in which sustained elevated IFN (and associated gene expression) promotes an immunological environment that is not conducive to development of activated B cells. Additionally, the findings of Campillo-Gimenez and Cumont are discussed in the Introduction and Discussion at lines 76-81 and 292-295.

**Reviewer #2**

*1. Lack of seropositivity for antibodies against the virus envelope in rapid progressors SIV-infected nonhuman primates was previously described in several other studies (Ex Pauza et al., Brocca-Cofano et al. etc) and this should be cited and discussed in the current paper. This will strengthen the findings in the current study, as it seems that lack of antibody production is the strongest criteria to differentiate the animal groups in RP and TypP. The survival of the two groups is not used to differentiate the two groups. It looks like the lack of antibody production is a common feature for rapid progression in both young and adult SIV-infected nonhuman primates, belonging to different species and this was reported by several studies.*

This is a good point that we have now written in greater detail in the manuscript. While we had previously cited the study by Brocca-Cofano et al., we have added Pauza et al. and now discuss the findings of both with the necessary detail. Indeed, lack of seropositivity has been previously associated with rapid progression in macaques, as well as human infants. New text is on lines 81-84.

*2. The possibility that the antibodies may in fact be produced in the rapid progressors but complexed by the higher levels of antigens produced by the rapid progressor and therefore may be undetectable in this subgroup should be also discussed.*

This is an interesting possibility; we had considered but not included in the manuscript for a number of reasons. First, the plasma viral load only increases on average 10-fold, while the antibody levels go from an average of 7x10^5 ng/ml in TypP infants to 1.1 x10^3 in RP infants (~700-fold reduction). Second, some of the TypP infants have similar levels of plasma viral loads at weeks 10-12 post-SIV when compared to RP infants, while large differences in levels of anti-Env plasma IgG at this same time point. For example, A17154 (TypP) has a viral load of ~1.4E7 copies/ml at 10-weeks post SIV and ~1.1E6 ng/ml IgG, whereas A14203 (RP) has a viral load of ~1.1E7 copies/ml at 10-weeks post SIV and ~1.8E3 ng/ml IgG. Third, TypP infants have significantly higher levels of activated memory B cells as well as germinal centers, which play central roles in antibody production. These B cell populations would not be influenced by antibody-antigen complexes and yet there are clear differences that associated with antibody data.

Therefore, we have not uncovered any evidence that antibodies in the rapid progressor might possibly be complexed with higher levels of antigens in the RP infants. Because we do not have any evidence, we believe it is best to not include this discussion in the manuscript as it may confuse the reader. If we are missing something in this regard, please let us know.

*3. Is there a significant difference in survival between the two groups?*

This is a good question, and one we can only partially answer. We do have some data in this regard as in the first year of our study we followed the infants for 20-week post-SIV infection. We found that all of the RP infant macaques (3 out of 6) succumbed to simian AIDS in that time frame (2 severe wasting, 1 experiencing clotting and seizures). None of the TypP infant macaques died in this first year 20-week follow-up. In consultation with the veterinarians at the Washington Primate Center we opted to alter the study endpoint to 10 weeks, thus avoiding unnecessary suffering while still allowing us to get the critical data with regard we needed to identify the TypP and RP macaques and evaluate their immune and viral outcomes. This was a difficult decision, but we think it was the most ethical choice.

*4. The graphs that include all the animals (Ex SIV RNA, ratio CD4/CD8, % of CD80+ B cells would benefit by the usage of just two colors, one for RP and one for the TypP). It is very difficult to differentiate the two groups.*

The graphs have been adjusted to more clearly report our results using 2 colors. The graphs that we have changed are located at Figures 3A and 5B.

*5. Fig. 3 The number of animals in the CD4 graphs (Fig 3 c) is smaller than in Fig 3A and B. Was only a subset of animals used in this?*

Thanks for catching this. This discrepancy was due to an oversight in updating this figure. Additional animals and timepoints have been added to 3C. This has only changed the data in that there is now a lower p-value used to define significant differences between groups at Weeks 10-12.

*6. The authors mention a possible difference in the number of viral variants that infect RP and TypP and this may be pursued experimentally (through SGA) to get a definite answer. I understand, however, that the volume of samples taken from the young animals is very small and may have prevented the group from performing these experiments, as they measured numerous other parameters*

While the reviewer raises a good point, it is true that we were limited by sample volume in the infants. If we had sufficient sample we would have undertaken SGA and whole envelope sequencing. However, we do have confidence in the deep sequencing of V1V2, that has a high sensitivity, with our method yielding tens of thousands of reads which we were able to computationally parse down to dozens of unique variants in each macaque. Additionally, this approach allowed us to assess phylogenetic relationships between plasma virus and the hundreds of variants present in our SIV challenge stock.

*7. Fig. 8. The detail selected shows MX1 increase adjacent to the germinal center, so the MX1 expression and localization this should be described as “in proximity to the germinal center” instead of “in the germinal center”.*

This comment was useful in helping to clarify these points in the manuscript. In this revised version we are careful to distinguish when we are referring to MX1 expression within or proximal to the germinal center. Note that the graphs emphasize the MX1 within the GC, not proximal to it. We have focused on this measurement as MX1 is visibly excluded from germinal centers in the typical progressors, whereas it is present in GCs of RP macaques.

*8. Was there any MHC Class I allele responsible for the clinical outcome. From Table 1 it looks like the authors genotyped the animals but they do not comment on this.*

This is a good question. While a fraction of infants possessed MHC-1 alleles previously associated with reduced viral replication (3/14 have the B*008 haplotype) outcomes could not be explained by MHC-1 allele frequency. In the first submission this was alluded to in the Methods. In the revised manuscript the first section of the Results has been updated to discuss MHC data in more detail. Additionally, Table 1 has been resorted so TypP and RP macaques are listed together, rather than intermixed.
